# Body composition and metabolic consequences of antibiotics most frequently administered to newborns in intensive care units: an experimental study in healthy newborn rats

**DOI:** 10.3389/fmed.2024.1369797

**Published:** 2024-04-23

**Authors:** Enrique Segura-Cervantes, Javier Mancilla-Ramírez, Liliana Fernández-Urrutia, Susana González-Gallardo, Lourdes Mendoza-Gertrudis, Jasibe Valencia-Santaella, Norma Galindo-Sevilla

**Affiliations:** ^1^Departamento de Epidemiología, Instituto Nacional de Perinatología, Mexico City, Mexico; ^2^Escuela Superior de Medicina, Instituto Politécnico Nacional, Mexico City, Mexico; ^3^St. Luke Escuela de Medicina, Mexico City, Mexico; ^4^Departamento de Patología, Instituto Nacional de Perinatología, Mexico City, Mexico; ^5^Escuela de Medicina, Universidad Anáhuac, Oaxaca, Mexico; ^6^Departamento de Infectología e Inmunología, Instituto Nacional de Perinatología, Secretaría de Salud, Mexico City, Mexico

**Keywords:** antibiotics, body composition, newborn, animal model, neonate development

## Abstract

**Introduction:**

The increasing overuse of antibiotics in recent years has led to antibiotics being the most prescribed drugs for pediatric patients, and 72% of patients in the neonatal intensive care unit are treated with antibiotics. One effect of antibiotic use is the alteration of the microbiota, which is associated with metabolic disorders, including obesity.

**Methods:**

This experimental study in newborn rats compared the administration of ampicillin/meropenem (Access/Watch groups) at 100/10 μg/g every 12 h, cefotaxime 200 μg/g every 24 h (Watch group), and amikacin 15 μg/g every 24 h (Access group) versus saline solution as the control. Each antibiotic was adjusted to the required dosages based on weight, and the doses were administered intraperitoneally daily for 5 days to 10–14 newborn male rats per group. A comparison of the morphometric and biochemical parameters registered on day 28 was performed using ANOVA.

**Results:**

Amikacin had the largest effect on morphometric measurements, and low-density lipoprotein cholesterol, while cefotaxime had the largest effect on glucose and triglycerides, whereas ampicillin/meropenem produced the weakest effect on the measured parameters.

**Discussion:**

The administration of antibiotics in the neonatal stage can affect the body composition of rats as well as the lipid and carbohydrate serum levels. Future studies should evaluate the toxicity of antibiotics in immature neonatal organs and could help to improve therapeutic decisions and prevent the unjustified use of antibiotics in newborns, thereby reducing metabolic consequences.

## 1 Introduction

Childhood overweight and obesity are major health system concerns worldwide. The prevalence of overweight and obesity among children and adolescents aged 5–19 increased from 4% in 1975 to 18% in 2016 (>340 million children and adolescents aged 5–19). Approximately 38.2 million children under the age of 5 years had overweight or obesity in 2019 (1).

Antibiotic treatment in the early days of life has been associated with abdominal fat accumulation in humans over the subsequent 6–24 months (2, 3). Antibiotics can eliminate or inhibit bacterial growth when used to treat neonatal bacterial infections, such as pneumonia, sepsis, and meningitis (4).

Antibiotic use is globally high in neonatal intensive care units (NICUs), where this represents 25% of the drugs used, and even higher in premature neonates due to the increased risk for sepsis (5, 6). The expression of costimulatory molecules in the immune system of neonates is low, and the immunological memory is weak, which is associated with a higher risk of severe neonatal infection and sepsis from perinatal exposure to pathogens and a high risk of neonatal death (7).

The World Health Organization classifies the antibiotics used in NICUs based on the Access, Watch, Reserve (AWaRe) Essential Medicines List for Children and their use. The antibiotics for the first level of access, such as ampicillin and amikacin, belong to a restricted spectrum. Observation antibiotics, such as meropenem and cefotaxime, have an antibacterial broad-spectrum, and the reserved group refers to those used in treating multidrug-resistant infections (8).

Although antibiotic use is essential, this can be regulated and targeted by the isolation of bacteria in culture, antibiograms, and new lab techniques, such as nucleic acid sequencing, which allow rapid, highly sensitive, and specific identification of bacterial species (9). However, the recovery rate by positive blood culture remains as low as <17%, which prevents timely specific treatment (8), and each hour of delay in antimicrobial administration over the ensuing 6 h has been associated with an average decrease in survival of 7.6% in critically ill patients (10). Therefore, providing rapid targeted therapy to the causative pathogen is critical.

The antimicrobial mechanism of action, pharmacokinetics, distribution, elimination, toxicity, and side effects are characteristics of each antibiotic and should be considered during the selection process (11, 12). Recently, the understanding of how patient age influences observed effects has become particularly important. The antimicrobial action extends as a secondary effect to the intestinal microbiota of the infant, affecting the diversity of colonizing microorganisms, particularly by decreasing the presence of Bifidobacterium, an abundant microorganism during lactation, and promoting the profusion of Klebsiella and *Enterococcus* spp., which remain up to 24 months after exposure to antibiotics (13, 14). This effect has been associated with the subsequent development of obesity in childhood.

Most current studies do not identify the specific antibiotics associated with the neonatal microbiome and weight changes. However, the mechanism of action of each antibiotic varies (11, 12), and associating the type and magnitude of a side effect with the antibiotic used is important when choosing between treatment options. Moreover, the genetic background, feeding, and ambient variations in infants increase the difficulty of comparing the effects of different antibiotics under such heterogeneous conditions. Therefore, animal models offer the advantage of comparing the effects of antibiotics under similar controlled conditions.

Herein, to compare the side effects of antibiotics frequently used in NICU, we applied a newborn rat model in a controlled environment to evaluate the individual effects of three treatments preferably used in NICUs on morphometric and biochemical adult-equivalent changes.

## 2 Materials and methods

### 2.1 Ethical statement

The protocol for this study was evaluated and approved by the Research, Ethical, and Animal Care for Research Use (CICUAL) Committees of the Instituto Nacional de Perinatología (INPer), under the registration number INPer-212250-3100331.

### 2.2 Study design

**Figure 1:**
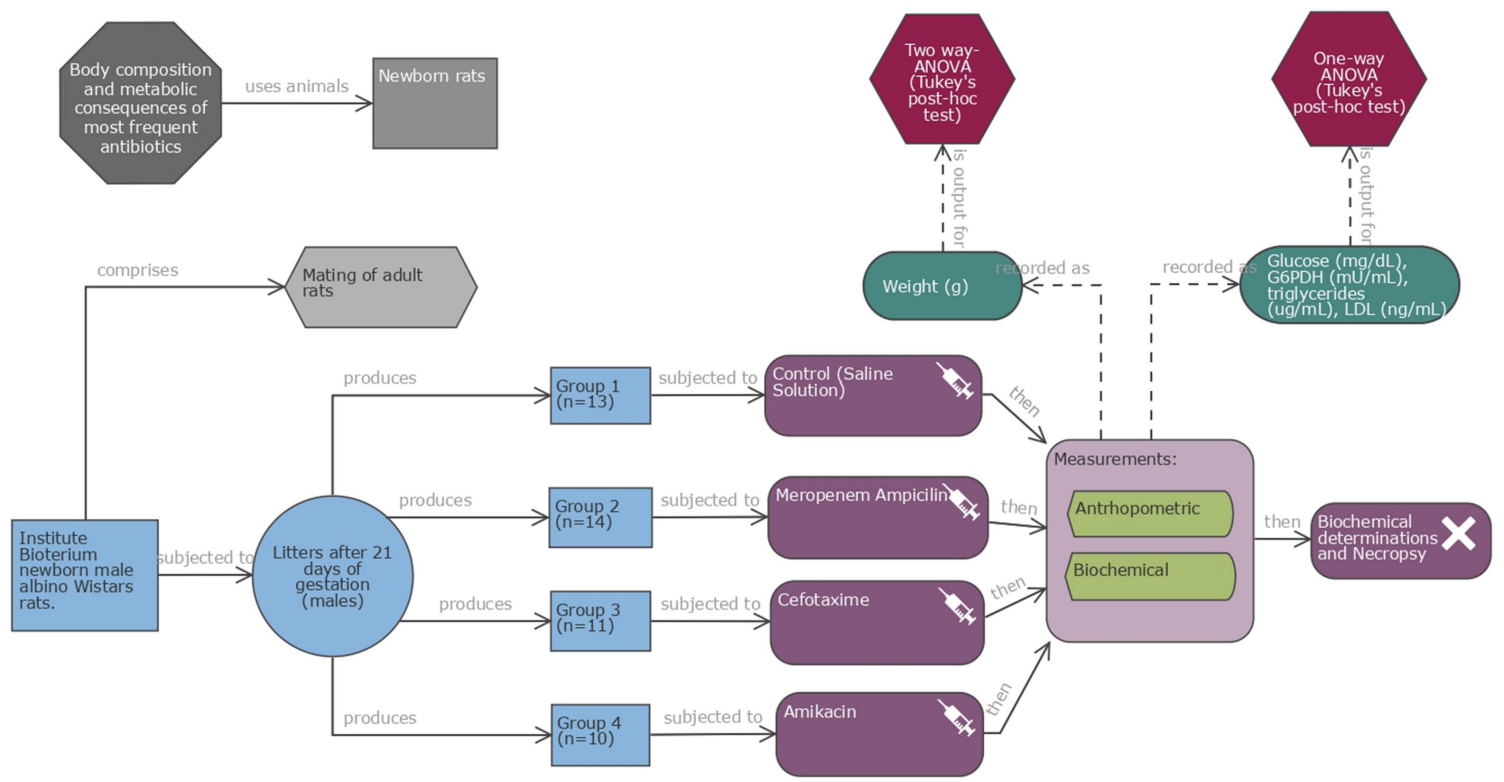
Schematic representation of the experimental study. Four groups of newborn male albino Wistar rats (10–14 each) were used: group 1 as the control C and three groups for treatment with altricial species antibiotics: ampicillin/meropenem (AW), cefotaxime (W), and amikacin (A), respectively. Antibiotic doses were administered intraperitoneally (IP) over 5 days. Rats were weighed weekly until the age of 28 days, when they were euthanized after 12 h of fasting. Blood was collected. (Diagram created using EDA digital tool http://eda.nc3rs.org.uk).

Healthy newborn male albino Wistar rats (1 day old, 6.4–7.5 g) were provided by the animal facilities of INPer. The rat litters were kept with the mother rats and fed on their mother’s milk in a temperature-controlled environment (22–24°C) with 55–60% humidity under a 12 h light–dark cycle for 7 days throughout the experiment. Newborn rats were randomly selected into four groups of 14 rats each to receive antibiotics administered intraperitoneally (IP) for 5 days, starting on the first day of life. Doses were prepared individually according to the rat’s weight (50 to 150 μL per dose). The rats were weaned at 21 days of age and weighed weekly from birth until the age of 28 days, when they were euthanized by halothane inhalation and cervical dislocation after 12 h of fasting. Blood samples were drawn immediately via cardiac puncture and separated into serum and whole blood cells. The serum samples were frozen at −70°C until use. Each rat underwent a necropsy. The study diagram is shown in Figure 1.

### 2.3 Antibiotic dosing

Four antibiotics were tested and adjusted to the required dosages based on each rat weight: ampicillin and meropenem (Access/Watch, group AW) in combination at 100/10 μg/g every 12 h; cefotaxime (Watch, group W), at 200 μg/g every 24 h and amikacin (Access, group A) at 15 μg/g every 24 h. Doses equivalent on weight basis to those administered to the newborns in the NICU were used. The main characteristics of these antibiotics are described in Table 1. In the control (group C), physiological saline solution was administered at a similar volume (μ/L) via IP every 24 h. Antibiotics administered every 24 h were injected via IP at 7 a.m., and those for every 12 h had an additional injection IP at 7 p.m.

**Table 1:** Antibiotic characteristics (from 11, 12).

Antibiotic	Meropenem	Ampicillin	Cefotaxime	Amikacin
Family	Carbapenems	Beta-lactams	Third generation cephalosporins	Aminoglycoside
Mechanism of action	Inhibits cell wall synthesis, binding to penicillin-binding proteins (PBPs) type 2, 3, and 4, thus facilitating bacterial lysis, bactericidal effect	Inhibits the last stage of cell wall synthesis by binding to the inactivating transpeptidase, and therefore preventing the cross-linking of the peptidoglycan chains that give the cell wall structure, causing bacterial lysis and subsequent death	Inhibits the last stage of bacterial wall synthesis by binding to PBPs, disrupting synthesis, and causing cell lysis	Binds to the 30S subunit of the bacterial ribosome, stopping bacterial DNA transcription and protein synthesis
Specific action	Gram-positive, Gram-negative, and strict anaerobes. Prescribed for complicated, intra-abdominal polymicrobial infections; also used in cases of meningitis.	Group b *Streptococcus*, *Listeria Monocytogenes*, and *Escherichia coli*	Gram-positive, Gram-negative, broad spectrum. *Streptococcus* spp. and Gram-negative bacilli. Has no activity against non-fermenting (*Pseudomonas aeruginosa and Acinetobacter baumannii*), *L. monocytogenes, Enterococcus* spp., or strict anaerobes	Gram-negative *Pseudomonas, E. coli, Proteus*, *Providencia*, *Klebsiella*, *Enterobacter*, *Serratia*, and *Acinetobacter* spp
Elimination	Renal elimination, including glomerular filtration and tubular secretion	Renal, glomerular filtration, and tubular secretion, with 10% hepatic metabolism	Renal elimination	Renal elimination
Toxicity		Interstitial nephritis, neurotoxicity, and platelet disturbance	Use has been associated with an increased risk of necrotizing enterocolitis	Risk of causing irreversible, bilateral, symmetrical ototoxicity
Newborn dose	Recommended dose 20 mg/kg every 12 h, for those under 32 gestational weeks and 30 mg/kg for those older than 32 weeks, every 8 h	The commonly used dose is 200 mg/kg/day (range 100–350 mg/kg/day every 6–12 h, extrapolated from adult studies). However by gestational age (GA) for premature babies, 50 mg/kg/dose every 12 h, or every 8 h if the baby is >34 weeks (GA)	Recommended dose 50 mg/kg/dose every 12 h for the first week of life	Dose according to weight of newborn 16 mg/kg every 36–48 h

### 2.4 Experimental animals

Rats’ weight was recorded daily on a calibrated electronic scale (AccuLab, VI-200, 11722, NY, USA). The rats were weaned at 21 days of age and remained in the cage when the mother was removed. The rats were allowed *ad libitum* access to food and water for an additional week and then euthanized at 28 days of age for biochemical parameters determination and necropsy. The rat ELISA kit (CP: 430223, CUSABIO, Wuhan, Hubei Province, China) was used according to the manufacturer’s instructions. Glucose (GL44 blood glucose monitor, mg/dL) and rat glucose-6-phosphate 1-dehydrogenase (G6PDH; Cat. No. CSB-EL009121RA) were reported in mU/mL, rat triglyceride (TG, cat. No. CSB-E11705r) was reported in μg/mL, and rat low-density lipoprotein cholesterol (LDL-C, Cat. No. CSB-E16561r) was reported in ng/mL.

### 2.5 Sample size calculation

Sample size calculation was performed based on the outcome of weight gain at 28 days from birth evaluated using the ANOVA model. A comparison between four study groups was considered with 95% reliability (*α* = 0.05), a statistical power of 80% (*β* = 0.20), and a grade effect size (Cohen’s *f* = 0.45). The minimum size of each study group was 14 experimental units. Sample size calculation was performed in R software version 4.3.2 with the “pwr” package.

### 2.6 Statistical analysis

Data were analyzed using GraphPad Prism v.9.1. Data are presented as means and standard deviations (SDs). Weight gain was calculated as the difference between the weight at 28 days and that at birth baseline. The comparison of weight means between study groups was performed each week from birth using a two-way ANOVA (antibiotics and age of the rat). *Post-hoc* tests were conducted with Tukey’s test for multiple comparisons, and the Benjamini, Krieger, and Yekutieli test (*q*-values) were used to control the false discovery rate (FDR). For comparisons of biochemical parameters and weight gain on day 28, a one-way Brown–Forsythe ANOVA (G6PDH and LDL) or a Kruskal–Wallis test (glucose and TG) was used. The model selection was based on residual analyses. Multiple comparisons between groups were performed using Dunnett’s T3 *post-hoc* test in the ANOVA models and Dunn’s *post-hoc* test in the Kruskal–Wallis models. The values of the biochemical parameters are plotted as box and whisker plots. Differences were considered significant where the *p*-value was <0.05.

## 3 Results

Initially, 56 rats were included in this study. During the pilot intraperitoneal administration of the first dose, the research team failed to execute the proper technique, causing the immediate death of eight rats. The study was restarted once the correct IP injection technique was achieved, preventing deaths attributable to this technical error in the experimental groups. A total of 13, 14, 11, and 10 rats were included in the control (C), ampicillin-meropenem (AW), cefotaxime (W), and amikacin (A) groups, respectively, with a total loss of eight rats. One-day-old male newborn rats were homogeneous in terms of morphometric measurements (Table 2). The weight values of each study group at birth baseline and at days 7, 14, 21, and 28 are shown in Table 3. Weight gain at 28 days was as follows: C: 47.38 g (SD: 6.17), WA: 52.63 g (SD: 2.93), W: 62.66 g (SD: 5.53), and A: 61.88 g (SD: 2.42). On day 28, the cefotaxime W (*p* < 0.0001) and amikacin A (*p* < 0.0001) groups showed significant differences compared with the control C group. However, no significant differences were observed between the control C and meropenem/ampicillin AW groups (*p* = 0.78). The cefotaxime-W (*p* = 0.002) and amikacin-A-treated groups (*p* < 0.0001) showed significant differences compared to the ampicillin/meropenem-treated group WA without significant difference from each other groups (*p* = 0.99).

**Table 2:** Baseline morphometric data of the newborn healthy rats.

	C group Control (*n* = 13)	AW group Ampicillin/Meropenem (*n* = 14)	W group Cefotaxime (*n* = 11)	A group Amikacin (*n* = 10)
Weight (g)	6.48 (0.43)	6.83 (0.47)	6.81 (0.96)	6.88 (0.43)
Rostro-caudal length (cm)	7.11 (0.23)	7.10 (0.24)	7.32 (0.41)	7.64 (0.32)
Thoracic diameter (cm)	3.97 (0.27)	4.21 (0.23)	4.05 (0.29)	4.16 (0.11)
Abdominal diameter (cm)	4.10 (0.28)	4.26 (0.30)	4.05 (0.25)	4.35 (0.12)

**Table 3:** Weight values of the rats in each study group during baseline, 7, 14, 21, and 28 days of follow-up.

	Control	A/M	Cefotaxime	Amikacin
Baseline groups	C	AW	W	A
Mean (SE)	6.48 (0.119)	6.84 (0.127)	6.81 (0.291)	6.88 (0.136)
*p*-values multi-test comparison				
vs. Control	–	0.79	<0.0001	<0.0001
vs. M/A	0.79	–	<0.0001	<0.0001
vs. Cefotaxime	<0.0001	<0.0001	–	0.17
vs. Amikacin	<0.0001	<0.0001	0.17	–
*q*-values for controlling FDR				
vs. Control	–	0.06	0.06	0.06
vs. M/A	0.06	–	0.08	0.08
vs. Cefotaxime	0.06	0.08	–	0.07
vs. Amikacin	0.06	0.08	0.07	–
7 days				
Mean (SE)	13.38 (0.181)	13.02 (0.255)	13.45 (0.480)	15.98 (0.226)
*p*-values multi-test comparison				
vs. Control	–	0.79	<0.0001	<0.0001
vs. M/A	0.79	–	0.0002	<0.0001
vs. Cefotaxime	<0.0001	0.0002	–	0.17
vs. Amikacin	<0.0001	<0.0001	0.17	–
*q*-values for controlling FDR				
vs. Control	–	0.06	0.07	0.002
vs. M/A	0.06	–	0.06	0.001
vs. Cefotaxime	0.07	0.06	–	0.003
vs. Amikacin	0.002	0.001	0.003	–
14 days				
Mean (SE)	23.97 (0.170)	24.26 (0.327)	26.19 (0.763)	29.81 (0.353)
*p*-values multi-test comparison				
vs. Control	–	0.79	<0.0001	<0.0001
vs. M/A	0.79	–	0.0002	<0.0001
vs. Cefotaxime	<0.0001	0.0002	–	0.16
vs. Amikacin	<0.0001	<0.0001	0.16	–
*q*-values for controlling FDR				
vs. Control	–	0.06	0.005	<0.0001
vs. M/A	0.06	–	0.007	<0.0001
vs. Cefotaxime	0.005	0.007	–	0.0003
vs. Amikacin	<0.0001	<0.0001	0.0003	–
21 days				
Mean (SE)	34.26 (0.751)	36.34 (0.771)	42 (1.419)	45.03 (0.544)
*p*-values multi-test comparison				
vs. Control	–	0.79	<0.0001	<0.0001
vs. M/A	0.79	–	0.0002	<0.0001
vs. Cefotaxime	<0.0001	0.0002	–	0.16
vs. Amikacin	<0.0001	<0.0001	0.16	–
*q*-values for controlling FDR				
vs. Control	–	0.005	<0.0001	<0.0001
vs. M/A	0.005	–	<0.0001	<0.0001
vs. Cefotaxime	<0.0001	<0.0001	–	0.001
vs. Amikacin	<0.0001	<0.0001	0.001	–
28 days				
Mean (SE)	55.51 (1.573)	59.72 (0.799)	67.40 (2.269)	68.77 (0.788)
*p*-values multi-test comparison				
vs. Control	–	0.78	<0.0001	<0.0001
vs. M/A	0.78	–	0.0002	<0.0001
vs. Cefotaxime	<0.0001	0.0002	–	0.17
vs. Amikacin	<0.0001	<0.0001	0.17	–
*q*-values for controlling FDR				
vs. Control	–	<0.0001	<0.0001	<0.0001
vs. M/A	<0.0001	–	<0.0001	<0.0001
vs. Cefotaxime	<0.0001	<0.0001	–	0.02
vs. Amikacin	<0.0001	<0.0001	0.02	–

Data are presented as mean and standard error. Comparisons were conducted using the two-way ANOVA, *post-hoc* test using Tukey’s test, and Benjamini, Krieger, and Yekutieli test.

Table 3 shows the overall effects of treatment with various antibiotics during the follow-up period. The multiple comparison test in the ANOVA model showed that weight at all weeks was higher in the cefotaxime and amikacin groups compared to the control group, and the ampicillin/meropenem group did not show differences from the control group. As shown in Table 3, the FDR test (*q*-values) suggested that all study groups differed from the control group on days 21 and 28.

**Figure 2:**
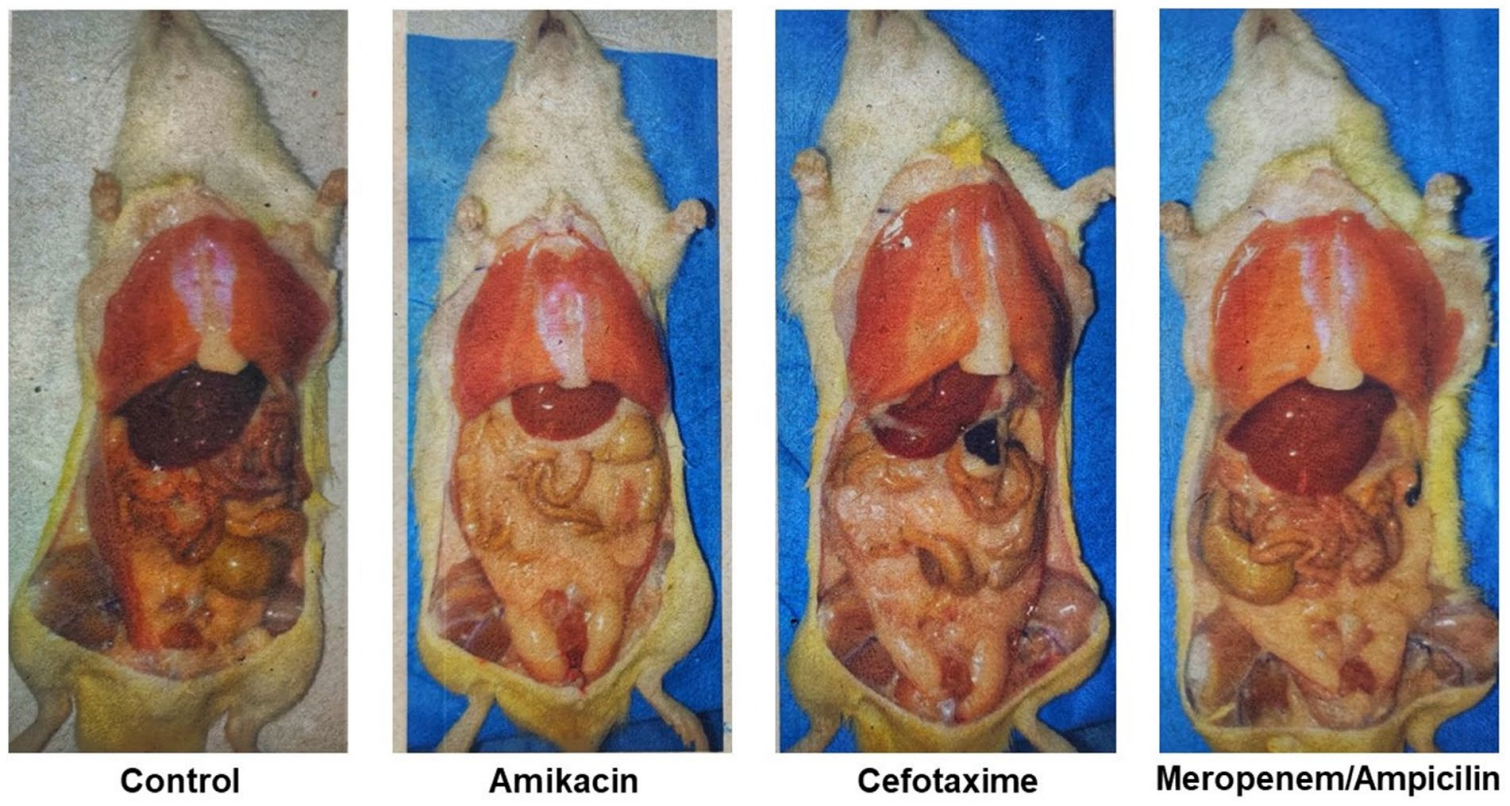
Abdominal fat accumulation in 28-day-old representative animals, each from a different group. Rats that received Cefotaxime or Amikacin showed a greater accumulation of fat.

The accumulation of abdominal fat at 28 days of age is presented on representative animals in Figure 2, which shows that fat accumulation was higher in rats treated with antibiotics compared to control.

**Figure 3:**
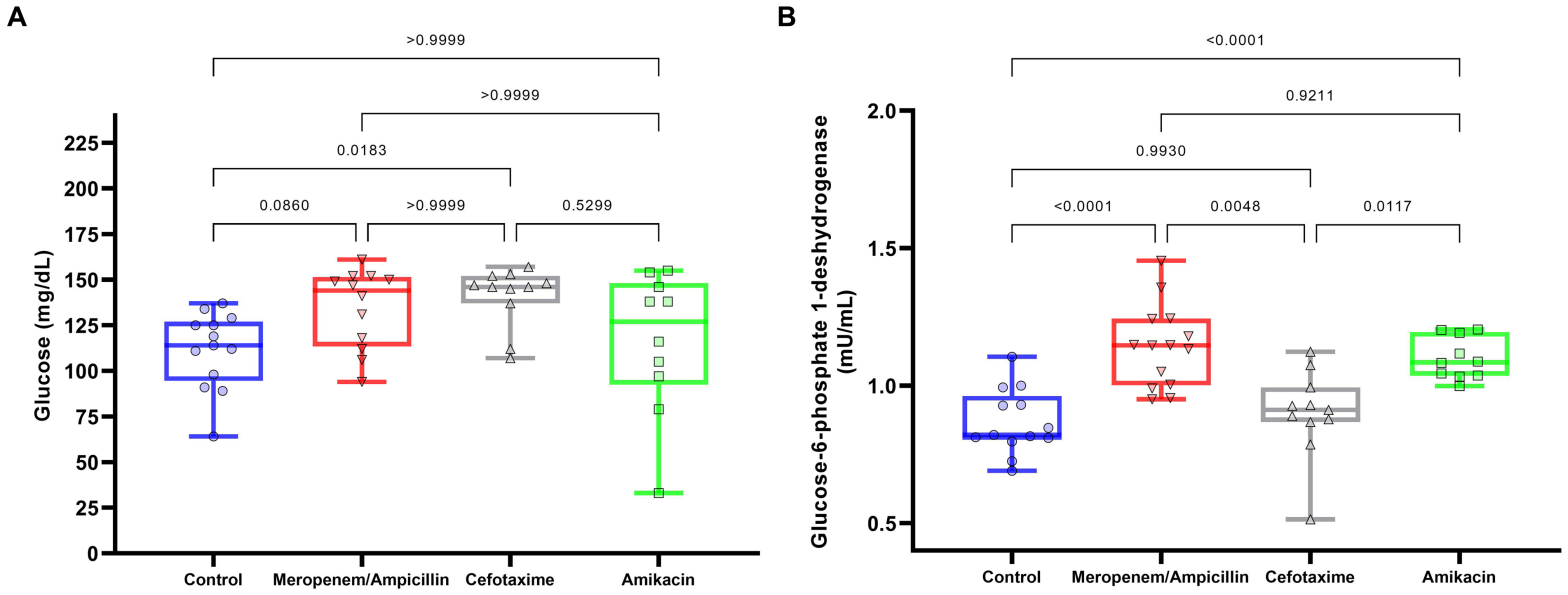
Effect of antibiotics on glucose and G6PDH levels in 28-day-old rats. **(A)** Glucose; **(B)** G6PDH. The box represents the median and first and third quartiles. Whiskers represent minimum and maximum values. Each point, triangle, or square represents a rat. Data are presented as boxes (25th, 50th, and 75th percentiles) or whiskers (minimum and maximum). Comparisons were conducted using the one-way ANOVA (G6PDH) and Kruskal–Wallis (glucose) models, *post-hoc* test using Dunnett’s T3 (G6PDH), or Dunn’s *post-hoc* test (glucose).

**Figure 4:**
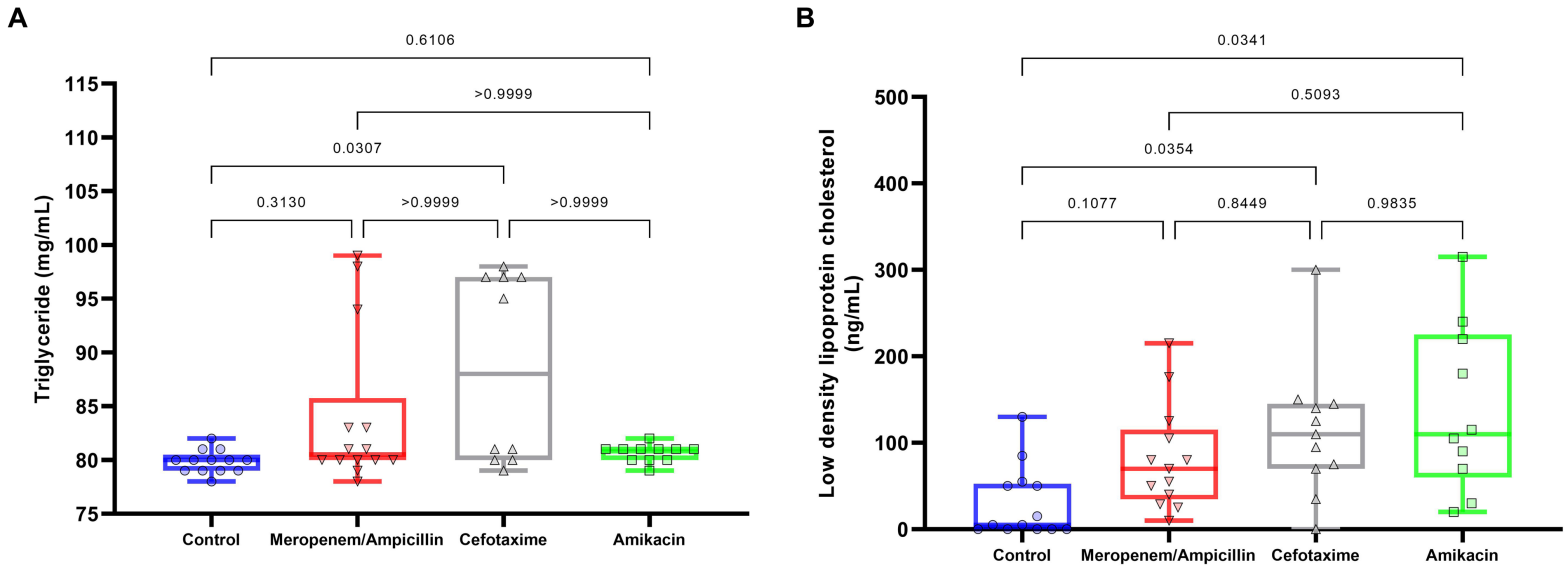
Effects of antibiotics on triglycerides and LDL values in 28-day-old rats. **(A)** Triglyceride levels; **(B)** LDL cholesterol values. The box represents the median and first and third quartiles. Whiskers represent minimum and maximum values. Each point, triangle, or square represents a rat. Data are presented as boxes (25th, 50th, and 75th percentiles) or whiskers (minimum and maximum). Comparisons were conducted using the one-way ANOVA (LDL) and Kruskal–Wallis (TG) models, *post-hoc* test using Dunnett’s T3 (G6PDH), or Dunn’s *post-hoc* test (glucose).

Distributions and comparisons of the biochemical parameters between the groups are shown in Figures 3, 4; levels of glucose and G6PDH on day 28 are shown in Figures 3A,B. Glucose levels were similar between the different study groups (control: 111.38 [SD: 20.93], ampicillin/meropenem: 134.42 [SD: 21.76], cefotaxime: 140 [SD: 16.38], and amikacin: 116 [SD: 38.83]). A significant difference was observed only between the cefotaxime group and the control (*p* = 0.02).

However, the levels of G6PDH, an important enzyme for the catalysis of NADPH production that protects cells against oxidative stress, exhibited significant differences between groups; control C: 0.87 [SD: 0.12], ampicillin/meropenem AW: 1.14 [SD: 0.15]; cefotaxime W: 0.899 [SD: 0.16]; and amikacin A: 1.1 [SD: 0.08], with the levels in the ampicillin/meropenem and amikacin groups being greater than those in the control (both: *p* < 0.0001) and cefotaxime groups (*p* = 0.005 and *p* = 0.01, respectively). In reference to lipids (Figure 4), TG (Figure 4A) tended to be higher in the cefotaxime W (88.5 [SD: 8.8]) and ampicillin/meropenem (84 [SD: 7.2]) AW groups, and similar in the amikacin A (80.5 [SD: 0.8]) and control C groups (79.8 [SD: 1.07]). Only the cefotaxime W group exhibited a higher concentration compared to the control C group (*p* = 0.03).

Conversely, the levels of LDL (Figure 4B) increased with the use of cefotaxime group W (113 [SD: 77.9]; *p* = 0.04) and amikacin group A (138.5 [SD: 96.8]; *p* = 0.03) in contrast to the control group C (30 [SD: 41.2]) with no significant difference to the ampicillin/meropenem group AW (81.5 [SD:60.4]).

## 4 Discussion

In this study, we used an animal model to compare the effects of antibiotics frequently used in neonatal intensive care on metabolic and weight changes during the first month of life. We observed an increase in weight gain of antibiotic-treated rats at birth due to fat accumulation and deficient regulation of carbohydrates and lipids. This effect was not homogeneous for the antibiotics tested, which may have been caused by differences in their individual mechanisms of action, elimination, dosage, and toxicity patterns. This animal model allows some precision in our observations, which is otherwise difficult to achieve in a heterogeneous population, such as human patients. Differences in weight gain have been observed since the first week of life. According to the physical growth assessment curves of male Wistar rats with reference values of body weight (g) as a function of chronological age (days), the weight reached on day 28 of life did not exceed the 97th percentile, falling outside the obesity parameter for chronological age (15), but within the overweight range. Weight gain has been associated with changes in the microbiota in both human and animal models. Interestingly, rats in the ampicillin/meropenem group (AW) showed the lowest weight gain.

A study examining the microbiota after antibiotic treatment in young adult mice reported taxonomic changes in the microbiome, changes in the copies of key genes involved in carbohydrate metabolism, and alterations in the regulation of hepatic lipid and cholesterol metabolism (16). The gut microbiota modulates blood lipids, including cholesterol levels (17), which could explain why antibiotic use increases TG levels in cefotaxime- (W) and ampicillin/meropenem- (WA)-treated groups. This effect was not observed in amikacin-(A)-treated rats, possibly due to differences in the sensitivity of the main bacteria in the rat’s microbiota to this antibiotic. Despite the significant differences in TG levels, the range of values was comparable with that reported in a previous study (18), although the lipid serum levels in that study were measured in adult rats.

LDL cholesterol levels increased in the amikacin- and cefotaxime-treated groups and were considerably different from those in the control group. The values observed in the control group in our study were comparable with those reported in a previous study (18), indicating that large differences in the antibiotic-treated groups are likely to be clinically relevant. The long-term trajectories of lipids after antibiotic treatment should be studied to characterize their clinical relevance.

Antibiotics have varying effects on the intestinal microbiota and consequently influence glucose and lipid metabolism. We hypothesized that nephrotoxicity and hepatotoxicity could also affect metabolism (19, 20). This is a starting point for further investigations of organic toxicity, possibly via biochemical studies utilizing toxicity markers and histopathological examinations. Notably, certain antibiotics exert their effects through alternative pathways, such as orexigenic mechanisms, with macrolides serving as an example (21). Although no macrolide drugs were used in this study, their consideration holds significance for future investigations.

The concentrations of each antibiotic we tested were different because the recommended therapeutic doses were distinct. The lowest concentration used in this study was for amikacin, which also adhered to the recommended dose. Amikacin did not affect TG or glucose levels, perhaps revealing that the other antibiotics (WA, W) affected the metabolism of carbohydrates and lipids. Thus, the observed effects could be attributed to organic toxicity caused by the antibiotics themselves or, more unlikely, by the components used to make them soluble.

Furthermore, these effects have been associated with well-documented antibiotic-induced changes in the intestinal microbiota and mitochondrial damage, potentially leading to antibiotic-induced obesity (22, 23). Importantly, most organs are still developing at the time of treatment. In particular, in a rat model with multiple gestations and immature breeds at birth, short gestation, rapid maturity, and short adult life (known as altricial species), organs are underdeveloped at birth. For example, pancreatic β-cell neogenesis continues throughout neonatal life. On postnatal days 1–2 the total islet mass doubles; on days 4–10 the rate of islet neogenesis is higher than the rate of islet growth, resulting in the pancreas being fully developed (24). Compared to humans, changes in pancreatic maturity in rats occur in the last weeks of pregnancy (25), with the pancreas expanding during the neonatal period (26). Prematurity disrupts normal pancreatic development by precipitating events that cause the incomplete expansion of pancreatic β-cells and a lower number of islets, making it difficult for the pancreas to respond to the demand for glucose regulation later in adulthood. In this comparison, the experimental period of this study corresponded with human prematurity, meaning that the use of antibiotics during this period may increase the risk of developing diabetes, although this depends on the type of antibiotic used. Despite the significant differences among the groups, none of the observed mean values were in the diabetes range (>200–300 mg/dL) (27, 28).

According to the equivalence parameters between human and rat lifespans (29), the antibiotic administration time in this study was longer than usual, and the follow-up time was relatively short, corresponding to close to 2 years of human life. Most studies that associate antibiotic treatment with metabolic changes do not distinguish between the gestational ages of infants during treatment. When full-term neonates were studied for their effect on weight gain after antibiotic treatment in the neonatal period, researchers found no weight gain even after transplanting the microbiota of treated babies, who also lost weight (30).

Importantly, results of testing for FDR showed differences with respect to the multiple comparisons of weights throughout the follow-up of the study, indicating that the weights between study groups at baseline and at 7 days were similar between study groups, whereas, from 21 days onward, all groups were different from each other. The cefotaxime W and amikacin A groups had the highest weight values. The use of *q*-values is more useful in this type of statistical analysis, in which multiple comparisons are conducted to avoid committing a type 1 error.

A limitation of this study is the lack of quantitative fat measurements when the animals were necropsied. Another minor limitation of our study was the loss of eight rats during the intraperitoneal administration of the first dose, which prevented the minimum calculated sample size from being obtained. Nonetheless, statistical evaluations were conducted for each model to ensure the adequacy of the model assumptions for comparisons. Additionally, we observed that since the study was conducted on healthy rats, the effect of antibiotics was not replicated exactly, as this occurs in neonates in the NICU due to multiple variables. Consequently, the study results cannot be extrapolated to neonates in the NICU because the variables were not the same. Therefore, this experimental study only compared the effects of antibiotics on the studied parameters.

Knowledge of metabolic alterations in newborns during antibiotic treatment is important. Future experiments could be directed to mitigate circumstances like these: (1) restoring normal microbiota as soon as possible after antibiotic treatment; (2) providing protective substances for organ development, such as those of lipid origin (vitamin E) used to protect neonatal rats from amikacin (31); and (3) prioritizing the use of antibiotics with lower therapeutic effective doses to minimize damage, thereby trying to protect postnatal developing organs. In adults, several FDA-approved drugs modulate body composition and metabolism. For example, steroid hormones such as glucocorticoids, which are used as anti-inflammatory agents, can stimulate weight gain and lower metabolism. Antibiotics can also have transient effects that typically result in weight gain. Additionally, other drugs, such as antidepressants, antipsychotics, antiepileptics, antihyperglycemics, and beta-blockers, play significant roles in regulating body composition and promoting weight gain (32–36). Although several FDA-approved drugs, such as antidepressants, are not commonly used in newborns, antibiotics and anti-inflammatory drugs are frequently administered. The effects of these two drug groups, especially on premature infants, require further study, as demonstrated by the findings of this study.

The impact of antibiotics on food-producing farm animals must be considered this imposes a positive pressure on the emergence of antimicrobial-resistant (AMR) bacteria (37). Furthermore, AMR genes can be transmitted to humans through the consumption of meat-harboring resistant bacteria, as in the case of ESKAPE bacteria (*Enterococcus faecium*, *Staphylococcus aureus*, *Klebsiella pneumoniae*, *Acinetobacter baumannii*, *Pseudomonas aeruginosa*, and Enterobacter species), where pigs are one of the biggest reservoirs of AMR (38).

Experimental studies in animal models as well as in humans have shown that different types of antibiotics may influence the development of obesity among children and that they can subsequently alter the human gut microbiota (obesity-related dysbiosis) (39–42). Therefore, careful selection of certain broad-spectrum antibiotics in children may reduce the burden of obesity and gut microbiota-related diseases.

Several issues could not be addressed in this study, such as the reversibility of the observed effects, the timing of damage, the dose-dependence of damage, and the impact of antibiotics on postnatal organ development compared to the effect on the microbiome. Most importantly, obesity not only results from an imbalance between calorie intake and expenditure but also is triggered by external events, such as the preconception of overweight of the mother.

In conclusion, the weight gain observed in children under antibiotic treatment in the first days after birth was confirmed in a healthy infant rat model, with values in the overweight range but not in the obesity range. The weight gain was lower with ampicillin/meropenem treatment than with amikacin or cefotaxime treatment alone. Increased glucose levels that did not meet the criteria for diabetes were found in the cefotaxime group compared to those in the control group. Large increases in LDL levels were observed in the cefotaxime and amikacin groups compared to the control group, which were larger than those reported in previous studies. Overall, these changes could be mediated by mitochondrial dysfunction, interference with normal organogenesis, or the early life microbiome, which are known to have long-term impacts on weight and metabolic parameters (22, 23). Therefore, it is necessary to conduct further and closer observations on the effects of antibiotics on metabolism. This research will help develop precise protocols for the selection of antibiotics for use in the NICU, aiming to minimize metabolic consequences and the risk of obesity and diabetes later in life.

## Data availability statement

The original contributions presented in the study are included in the article/supplementary material, further inquiries can be directed to the corresponding author.

## Ethics statement

The animal study was approved by “*Comité de Investigación, Ética y CICUAL del INPer*” under registration number 212250-3100331. The study was conducted in accordance with the local legislation and institutional requirements.

## Author contributions

ES-C: Writing – review & editing, Project administration, Funding acquisition, Conceptualization. JM-R: Writing – review & editing, Formal analysis. LF-U: Writing – original draft, Formal analysis. SG-G: Methodology, Writing – review & editing. LM-G: Writing – review & editing, Methodology, Investigation. JV-S: Writing – original draft. NG-S: Writing – review & editing, Writing – original draft, Formal analysis, Conceptualization.
